# Genotyping by Sequencing Using Specific Allelic Capture to Build a High-Density Genetic Map of Durum Wheat

**DOI:** 10.1371/journal.pone.0154609

**Published:** 2016-05-12

**Authors:** Yan Holtz, Morgane Ardisson, Vincent Ranwez, Alban Besnard, Philippe Leroy, Gérard Poux, Pierre Roumet, Véronique Viader, Sylvain Santoni, Jacques David

**Affiliations:** 1 Montpellier SupAgro, UMR AGAP, Montpellier, France; 2 INRA, UMR AGAP, Montpellier, France; 3 INRA, UMR 1095, Genetics, Diversity and Ecophysiology of Cereals, Clermont Ferrand, France; 4 UBP, UMR Genetics, Diversity and Ecophysiology of Cereals, Clermont Ferrand, France; Montana State University Bozeman, UNITED STATES

## Abstract

Targeted sequence capture is a promising technology which helps reduce costs for sequencing and genotyping numerous genomic regions in large sets of individuals. Bait sequences are designed to capture specific alleles previously discovered in parents or reference populations. We studied a set of 135 RILs originating from a cross between an emmer cultivar (*Dic2*) and a recent durum elite cultivar (*Silur*). Six thousand sequence baits were designed to target *Dic2* vs. *Silur* polymorphisms discovered in a previous RNAseq study. These baits were exposed to genomic DNA of the RIL population. Eighty percent of the targeted SNPs were recovered, 65% of which were of high quality and coverage. The final high density genetic map consisted of more than 3,000 markers, whose genetic and physical mapping were consistent with those obtained with large arrays.

## Introduction

Wheat (*Triticum* spp.) is one of the most widely grown food grain crops in the world and provides about a fifth of the calories consumed by humans (FAO, http://faostat3.fao.org/home/E). Durum wheat [*Triticum turgidum* (L.) subsp. *turgidum* (L.) convar. *durum* (Desf.)] accounts for about 10% of the total global wheat production (World Grain Statistic, www.igc.int). It is a minor crop compared to bread wheat, which is the focus of considerable efforts with regard to breeding new high performance cultivars, genomic investigations and resource developments. In this respect, new omics resources developed under the umbrella of the International Wheat Genome Sequencing Initiative (IWGSC) represents a major input for all Triticeae genomic approaches [1]. Durum wheat (*Triticum turgidum* subsp. *durum*) is a modern representative of a group of allotetraploid subspecies (*Triticum turgidum* subsp.) that were domesticated from the wild *T*. *turgidum dicoccoides* [2,3]. It is closely related to bread wheat (*Triticum aestivum* L.), which arose via spontaneous interspecific hybridization between a domesticated *T*. *turgidum* spp. form (AB genomes, 2n = 4x = 28) and the wild diploid *Ae*. *tauschii* (D genome, 2n = 14) [4,5].

As durum wheat and bread wheat share two closely related genomes, molecular tools and basic research are mostly focused on bread wheat and are secondarily used for durum wheat breeding [6]. Molecular tools are now crucial for rapid and efficient breeding [7], so genotyping tools are constantly studied and being improved. Numerous durum wheat genetic maps have already been developed as described below. The number and type of markers used to build these genetic maps have both evolved rapidly over the last decades. The first genetic maps have been built with a few hundred loci based on restriction fragment length polymorphism (RFLP) markers or amplified fragment length polymorphism (AFLP) markers [8,9], In the early 2000s, the development of PCR markers such as SSRs, EST-SSRs, or DArTs [9–13] led to an increase in molecular resources while streamlining protocols. These new resources, which are often developed for the most important economic species (e.g. bread wheat), have therefore facilitated genotyping of secondary crops such as tetraploid wheat. Map resolution has thus been improved [10,13–15], while facilitating the delimitation of chromosome regions involved in the control of agronomical traits [16], and relevant comparisons of local recombination rates have been published [17]. Moreover, bridging information from different single cross maps has allowed high density consensus maps to be built based on several thousand markers. These have improved genome coverage, proposed a validation of marker ordering, and reduced large gaps due partly to the absence of polymorphism between parents [18–20].

Single nuclear polymorphisms (SNPs) in coding and noncoding sequences have recently become favorable markers for building high-density genetic maps thanks for their high abundance in the genome [16,21]. Rapid advances in sequencing capabilities and dramatic cost reductions have facilitated genome-wide discovery of SNPs, even for polyploid species such as wheat [22]. The reduction of genomic complexity via techniques such as RNAseq sequencing can help generate large SNP databases for bread wheat [23] and durum wheat [24]. These techniques can also be used for designing large-scale DNA microarrays for wheat [25] or for genotyping by sequencing [24]. But few specific durum wheat tools are currently available. For example, although durum wheat polymorphisms have been used on the recent wheat 90K iSelect array http://wheatgenomics.plantpath.ksu.edu), most polymorphisms were of bread wheat origin [6]. Nevertheless, such arrays can only reveal polymorphisms documented on large panels encompassing broad temporal (old and elite cultivars) and geographical diversity. When used on durum wheat, they generated good results for building maps, but few studies have dealt with elite x elite crosses [26]. Moreover, as such arrays are primarily focused on genotyping elite material, they may lack some polymorphisms specific to wild and ancient germplasm, such as the wild *T*. *turgidum* subsp. Lastly, this technology is remarkably cost efficient per data point for projects requiring a high number of polymorphisms, such as GWAS studies, but it remains expensive for genetic mapping, diversity surveys or genomic prediction programs when interesting SNPs have been identified. Medium throughput technology built on parental specific polymorphism may represent a valuable alternative to high throughput microarrays for several applications.

Array uses fluorescent technology, which also limits data interpretation, especially in a polyploid context with closely related sequences, like homeologous genes, where intralocus heterozygous states can be easily confused with interlocus divergence. Genotyping by sequencing thus appears to be a promising alternative and could be used even for *de novo* genotyping of large populations at low cost [27]. A first set of technologies is based on an efficient method involving genome complexity reduction combined with multiplex sequencing. Restriction associated DNA tagging (RAD) [28], Genotyping-by-sequencing (GBS) [29] or double digestion RAD (ddRAD) [27] target the genomic sequence flanking restriction enzyme sites to produce a reduced representation of the genome. However, GBS does not enable targeting of specific sequences in the genome and always encounters difficulties when dealing with complex polyploid genomes such as durum wheat presenting homeologous loci and many repetitive sequences [30].

Sequencing transcribed portions of the genome using RNA extracted from standardized tissues (RNAseq) is also a good alternative since the transcribed gene-coding regions represent only one to two percent of the whole genome [31]. Genotyping by sequencing large populations using RNAseq is feasible but, unfortunately, transcriptomic libraries are more costly than genomic libraries and this method leads to a high rate of missing data due to the presence of low expressed genes and the dependence of some gene expression on specific tissue or environmental conditions [24].

Whole exome capture [32,33] was shown to be an effective strategy to reduce genome complexity. It paves the way to genotyping by sequencing of complex genomes [22] and to discovering a tremendous number of gene polymorphisms. This approach requires a good reference genome to map the reads properly, enabling a proper calling of allelic intralocus variations, exploiting the divergence between homeologous gene copies [22]. This approach is now possible since large resources are available for wheat genome [1], in addition to the durum wheat transcriptome [24]. Whole exome capture (WEC) is a very powerful approach but is still costly when coverage of the whole genome is not needed.

In this case, reducing the number of captures to a small set of targeted polymorphisms is an efficient *ad hoc* genome reduction technique, notably in cases where polymorphisms have already been identified in a set of parental lines. Baits are synthesized from a documented set of SNPs and hybridized to genomic DNA for capturing relevant homologous fragments that are subsequently sequenced. Baits would also capture fragments highly homeologous and/or paralogous to the targeted sequence. The resulting reads could be nevertheless assigned properly and could permit the disclosure of untargeted SNPs.

Specific capture ensures that the population will be polymorphic at most targeted positions, once a given sequencing error level is accepted. Deep sequencing of relatively few targets (~5000) should also ensure a good coverage of those targeted loci, thus reducing the risk of missing data. This technique appears to be especially well adapted for the construction of dense genetic maps since the bait design can maximize the number of targeted contigs, as sequencing will reveal all SNPs in the contig area for which the bait has been designed. Targeting specific loci permits spreading of future markers along chromosomes, thus maximizing the detection of recombination events, which is a desirable feature for genetic map building. The density of the bait design can be very easily adapted in regions of interest (e.g. higher density nearby known QTL). Finally, sequence knowledge around an SNP eases the design of competitive allele-specific PCR markers (KASPar) (http://www.kbioscience.co.uk), which are attractive, cost effective and easy to use markers for breeders in routine breeding programs [34,35].

Here we report results of sequencing fragments captured with specific allelic baits applied on a durum wheat bi-parental segregating RIL population. The bait design is precisely described. We analyzed the capture efficiency and identified some caveats inducing the capture of off-target repetitive microsatellite sequences likely due to a daisy chain effect [36].

Genotyping by sequencing data has been successfully used to build a high density genetic map comparable to recently published maps using the 90K iSelect array. Marker positions are very consistent with those provided by the International Wheat Genome Sequencing Consortium (IWGSC, http://www.wheatgenome.org) on the bread wheat reference [1,37]. Some specific features are discussed as well as the advantages of this genotyping by capture approach.

## Material

The *Dic2* x *Silur* mapping population (DS) used in this study consisted of 135 F_6_ recombinant inbreed lines (RILs) derived from a cross between an emmer wheat accession (*Triticum turgidum* ssp *dicoccum*) named *Dic2* and the durum elite *Silur* variety.

A durum wheat *de novo* assembly of the transcriptome (DWr) composed of 80,691 contigs was available from a past study [38] (S1 File). Briefly, RNAs of 172 lines of an Evolutionary Pre-breeding pOpulation (EPO) were sequenced and assembled. Homeosplitter software (http://bioweb.supagro.inra.fr/homeoSplitter) was applied on this *de novo* assembly to unravel homeologous copies that were initially erroneously merged in a single chimeric contig [38]. Read alignments on this reference led to 84,710 high quality SNPs [24].

Transcripts of the bread wheat chromosome survey sequence for cv. Chinese Spring (BWr) generated by IWGSC and provided by the Ensembl database (http://plants.ensembl.org/Triticum_aestivum) was used here for comparison purposes [1,37]. All BWr contigs were attributed to a specific chromosome. The physical positions for chromosome 3B are available [39] and proxies for physical positions within other chromosomes were estimated using population sequencing (POPSEQ) data generated by Chapman et al. [40] (Ensembl release 28). An alternative estimation of the genetic positions of these contigs is also available from the IWGSC genome zipper [37].

DWr contigs were blasted against BWr. It gave us a putative assignment of DWr contigs to bread wheat chromosomes and provided putative physical [40] and putative genetic positions [37] of the DWr contigs and consequently of the carried SNPs.

## Method

### Initial polymorphism detection

RNA was extracted and purified for the two parents of the DS population and 18,899 SNPs were detected using the protocol of [24]. In brief, sequencing was carried out using the Illumina mRNA-Seq paired-end indexed protocol on an HiSeq2000. Thirty-eight and 41 million read pairs were produced for *Dic2* and *Silur* (resp.) and preprocessed with *Cutadapt* [41] to remove adaptor sequences, trim read extremities with low quality scores (parameter -q 20) and keep reads with a minimum length of 35 bp. We first mapped the cleaned reads on DWr using BWA [42] while allowing 3 errors (-n 3 in the aln step). We then used Picard tools (http://broadinstitute.github.io/picard/) to remove PCR and optical duplicates [43]. Remaining unmapped reads (23 million [64%] and 15 million [39%] for *Dic2* and *Silur* resp.) were then mapped on BWr using the same protocol, thus allowing us to map 3 and 2 million reads, respectively. *Reads2SNP* [44] was used for the genotype calling (*Fis* value = 0.8). Genotypes called with less than 10 reads or a *read2SNP* probability of below 99% were considered as missing data. Polymorphism was considered reliable only when both parents were homozygous, leading to 17,543 SNPs on DWr, and 1,356 additional on BWr (Table 1).

**Table 1:** Polymorphisms available for bait design.

*Available RNASeq SNPs from*	Number
** Dic2 x Silur**	
SNPs on DWr	17,543
SNPs on BWr	1,356
SNPs available (total)	18,899
SNPs targeted by baits	4,352
** EPO**	
SNPs available (total)	84,710
SNPs targeted by baits	1,888

### Bait design

Baits are 120 bp long sequences surrounding a targeted SNP. They were designed on single exons since baits spreading over multiple exons would not have complementary matches on the genome and would thus not efficiently capture the targeted sequence. Exon frontiers available from the BWr annotation [1] were used to annotate regions of interest for SNPs detected in DWr using a blast followed by exonerate via the TriAnnot pipeline [45]. Exon frontiers were detected for 59,922 of the DWr contigs (74%).

We considered only SNPs located in exons longer than 120 bp to be able to design single exon baits and we targeted only one SNP per contig to minimize the target redundancy. This strong filter reduced the number of targeted SNPs from 18,899 to 4,352. To complete this set of specific *Dic2* x *Silur* polymorphisms, 1,888 supplementary SNPs were chosen within the *EPO* dataset, with the same quality filters. This brought the total number of SNPs and consequently the number of contigs targeted in this study to 6,240.

**Fig 1:**
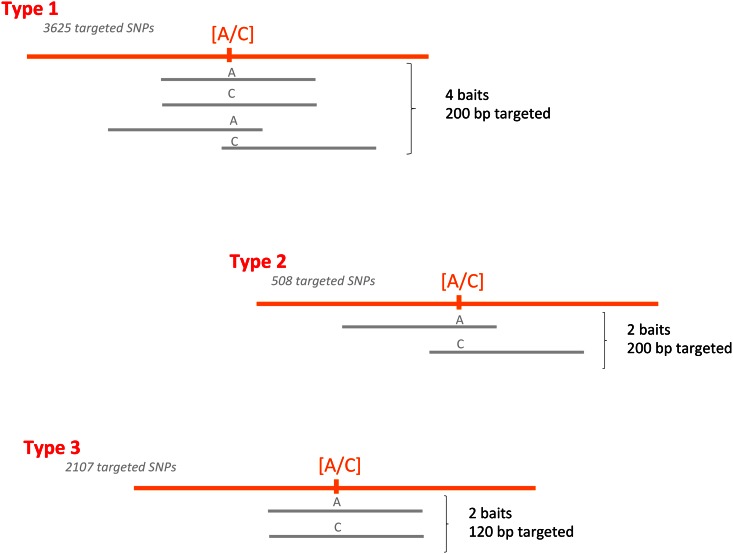
Description of the bait design. Orange lines represent the durum wheat genome, with the targeted SNP in brackets. Bait sequences are represented in grey. The number of SNPs targeted by each type of bait is specified.

Different bait design strategies were used. SNPs were targeted using two or four baits. As the first 3,625 SNPs were located in exons longer than 200 bp, four baits were designed for each: two centered on the targeted SNP, and two on its 5’ and 3’ flanking regions, hence targeting a total of 200 bp around the SNP (type 1). A second set of 508 SNPs also located in exons longer than 200 bp were targeted by two shifted baits only (type 2). The 2107 remaining SNPs were located in exons between 120 and 200 bp and targeted by two aligned baits (Type 3) (Fig 1). For each SNP, both *Dic2* and *Silur* alleles were used for probe design. The resulting 20,000 bait sequences were synthesized by MyBaits (http://www.mycroarray.com).

### Capture protocol

Briefly, for each member of the DS population, total DNA was extracted from fresh young leaves with the Chemagic DNA Plant Kit (Perkin Elmer). Library preparation for multiplexed individuals was done according to the protocol published by Rohland et al. [46]. Enrichment by capture is done with biotinylated RNA probes (120 mers) according to the manufacturer’s (MYBaits) protocol on pools of 48 barcoded genomic libraries (S2 File).

### SNP calling on captured sequences

Captured DNA was sequenced on an Illumina HiSeq3000, which gave paired reads of about 150 bp each. Reads were preprocessed and cleaned according to the protocol used for initial polymorphism detection. Mapping and genotype calling were done using whole DWr and targeted BWr contigs.

For targeted SNPs, as we were confident that polymorphism did exist, genotype calling was accepted with as few as two reads per individual. Recovered SNPs constituted the expected recovered SNP dataset (ER-SNP). *Read2SNP* identified new SNPs in loci homeologous or paralogous to the targeted one. A threshold of four reads per individual was used to call a genotype for these untargeted SNPs. These SNPs were likely in low expressed genes and were thus not detected in the preliminary transcriptomic analysis of the DS population parents. They accounted for the bonus SNP dataset (bonus SNP) that complemented the ER-SNP.

Finally, all SNPs were filtered according to three criteria so as to keep only highly reliable SNPs: i) a low number of heterozygotes controlled by an Fis value above 0.8, as 1.5% heterozygosity was expected after six successive selfing generations ii) at least 100 out of the 135 available RILs genotyped, and iii) balanced frequencies with a minimum expected heterozygosity (Nei’s He, [47]) of 0.34 so as to avoid segregation distortion, which is undesirable for genetic map building. This cleaning process led to the ER-clean-SNP and bonus-clean-SNP dataset.

### Map construction

All ER-clean-SNPs were used for the genetic map. Only one bonus SNP per contig on non-targeted DWr contigs was kept to avoid redundancy of genetic markers known to have strong DLs. The *mrkmerges* function of Carthagene [48] was used to merge all markers whose observed genotypes on all individuals were redundant. Initial linkage groups (LGs) were assembled using a LOD score ≥ 7 and a maximum two-point distance of 0.14. LGs were attributed to one of the 14 chromosomes according to the putative assignment (obtained by blast on BWr) of the majority of the SNPs of any given LG. Finally, the order and distance of markers within chromosomes were determined using the *build*, *annealing*, *greedy* and *flips* algorithms proposed by Carthagene. Markers of LGs attributed to the same chromosome were pooled for this final step.

In a second step, 249 additional SNPs for which the number of genotyped RILs was between 50 and 99 were positioned. The method is described in the S3 File and their position is given on the genetic map (S4 File).

To confirm the quality of the map, marker assignment to LGs were compared with putative assignment coming from BWr. Their orders and genetic positions on the genetic map were compared with putative physical and genetic positions in BWr. Spearman’s rank correlation coefficients were calculated on a per-chromosome basis.

For each chromosome, a third order polynomial regression was fitted between genetic and putative physical positions. This allowed us to identify SNPs whose predicted physical positions and mapped positions on DS were not consistent. Markers outside the 95% confidence interval of the model—although kept for further analysis—were tagged as outliers.

## Results

### Capture efficiency and SNP genotyping

A mean of 2.8 million (min: 0.4 M, max: 5.8 M) reads per sample was obtained. The cleaning step resulted in 2.5 million usable reads per sample on average (min: 0.3 M, max: 5.5 M), with an average of 0.3 million orphan reads.

Read mapping revealed a high degree of on-target enrichment efficiency for all accessions. Indeed, on average, 86% of the reads were mapped on DWr, which denoted a low degree of hybridization between baits and off-target DNA.

However, a high number of reads were accumulated on some microsatellite-like regions within contigs that were not carrying baits and 20 DWr contigs accounted for more than 42% of the total number of mapped reads. This phenomenon was due to the presence of microsatellite-like regions nearby the SNP targeted in these contigs, thus capturing thousands of small reads containing repetitive elements.

This means that some of our baits may have captured genomic fragments carrying microsatellites, even though none of our baits could have been considered as a “true” microsatellite. Close inspection of our baits revealed that very few of them (96) were carrying a 7-mer of dinucleotide repeated element or a 5-mer of trinucleotide repeated element.

Direct capture of long stretches of repetitive DNA such as microsatellite patterns by the baits seemed unlikely. The fact that we did not detect any other strongly repeated sequence in our reads suggests that the corresponding fragments were indeed captured primarily by our baits but successively amplified by a daisy-chain effect on non-targeted microsatellite capture. The daisy-chaining principle was first introduced in a cross hybridization context that used a standard adapter at the ends of each fragment of the genomic library. Blocking oligonucleotides must be used during the hybridization phase of the adapters to avoid these unexpected technical captures and reduce the adapter size [36,46]. We believe that in our case, we initiated a genomic daisy-chaining process among the multitude of wheat DNA fragments carrying microsatellite sequences. Although we could not be certain that we identified the “guilty” baits, we removed them from the bait database provided in the supporting information (S5 File). Using a blocking agent made of identified microsatellites might also reduce this daisy-chain effect and improve the target capture efficiency.

This huge presence of repetitive elements did not prevent capture of the targeted SNPs. Indeed, only 6% of the targeted positions were not covered at all. The median number of reads received per targeted contig per individual was 11.4.

Among the 6,240 targeted loci, 5,301 were thus recovered, but 768 of them designed on EPO were found to be monomorphic between *Dic2* and *Silur* (Table 2). A hundred and sixty (160 / 6,240) targeted SNPs only presented heterozygous individuals and were discarded. The remaining 4,373 ER-SNPs were then filtered according to their (> 0.8) Fis and (> 0.34) Nei’s He threshold, thus leaving 3,292 SNPs. From this set, 2,822 SNPs were genotyped for more than 100 individuals. These SNPs made up the ER-clean-SNP dataset. Finally, 45% of the total targeted SNPs were recovered. This success ratio would be as high as 65% if only polymorphic targeted SNPs based on *Dic2* x *Silur* parents are considered.

**Table 2:** Efficiency of the genotyping by capture protocol.

***SNP recovery***	
Number of targeted SNPs	6,240
**Not recovered SNP** (30%)	
Not captured	939 (15%)
Captured but monomorphic	768 *(12%)*
Heterozygote state only	160 (3%)
**Recovered SNP** (70%)	
OR-SNPs	4,373 *(70%)*
OR-clean SNPs (targeted and clean) (1)	**2,822 *(45%)***
** Bonus SNPs**	
Clean bonus SNPs (2)	2,305
On untargeted contig	1,410
Keeping only one SNP per contig (3)	**968**
** Total usable SNPs**	
Clean SNPs available (1+2)	5,127
SNP used for the genetic map (one per contig) (1+3)	**3,790**

**Fig 2:**
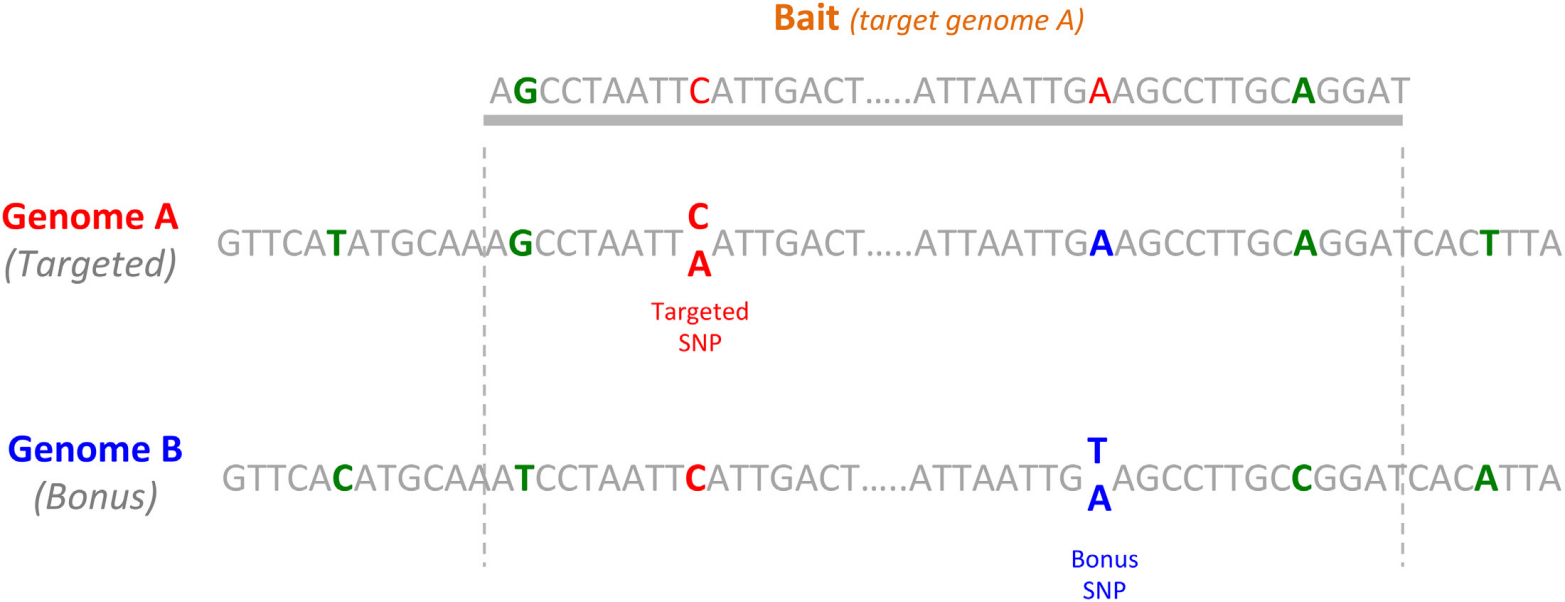
Detection of a bonus SNP on the homeolog of a targeted contig. A portion of A and B genomes were represented, with an SNP on the A genome (in red) and an SNP on the B genome (in blue). Divergences between both genomes are represented in green. The bait shown in grey was designed initially to capture a portion of the A genome, but captured also the homeologous portion of the B genome, with the related bonus SNP.

Many bonus SNPs were detected on positions that were not targeted by the baits. We identified 2,305 new SNPs passing the quality threshold filters. 895 (39%) of them belonged to targeted contigs and were thus discarded to avoid redundancy due to high linkage disequilibrium. The remaining 1,410 SNPs belonged to contigs homeologous or paralogous to the targeted ones (e.g. Fig 2). As for other contigs, only one SNP was kept per contig, leading to 968 clean bonus SNPs for the genetic map.

### Effect of the bait type

Three bait designs were tested. As expected, loci targeted using four baits (type 1) were about twofold more sequenced than those targeted using only two baits (bait types 2 and 3), which was consistent with the stoichiometric expectations. This variation in the average locus coverage, i.e. 8.53 reads (bait design 2), 9.02 (bait design 3) and 16.54 (bait design 1), had little impact on the proportion of ER-SNPs (ranging from 69.7 to 70.1%), but significantly impacted the average number of available genotypes per SNP (137.2 for type 1, 121.1 and 116.7 for types 2 and 3 resp.). The probability of an SNP passing the quality filter was thus impacted by the number of baits designed on one SNP: ~0.4 for two baits per SNP vs ~0.5 for four baits per SNP.

### Genetic map construction

Among the 3,790 SNPs, 56 were discarded (3,734 remaining) because *Dic2* and *Silur* alleles were unknown, and 1,624 unique positions were found by the Carthagene *mrkmerges* function. Only one SNP was kept per unique position for mapping and all markers at that position received the same genetic position (S4 File). Twenty-two linkage groups (LGs) were assembled. Fourteen LGs were constituted by more than 130 markers each, which was in line with the expectations since durum wheat has 14 chromosomes. Eight LGs had few markers (< 23 per LG). The LG 3 had only 2 markers and was not kept in the map. Three markers were not linked to any group and were not used in the genetic map.

Among the set of markers, 2,608 (68.5%) had a good blast hit on BWr and thus had a putative assignment to wheat chromosomes. Each LG was attributed to the chromosome that was attributed to most of its markers. The resulting putative assignments are provided in S1 Table.

### Map description

Table 3 summarizes the key genetic map features. Mapping positions of individual markers are given in the S4 file. The total map length was 2,964 cM, with an average chromosome length of 212 cM (range: 163.7 cM for chromosome 4B to 288.2 cM for chromosome 5A). The map length was evenly divided between the A (1527 cM) and B (1437 cM) genomes. The number of polymorphic sites was also evenly distributed, with 1,812 and 1,917 markers for genomes A and B, respectively.

**Table 3:** Features of the DS durum wheat genetic map.

Chr.	SNPs	Length (cM)	Intermarker distance cM/marker	Biggest gap (cM)	unique pos.	Spearman r with gen. pos.	Spearman r with phys. pos.
1A	231	175.5	0.76	18.2	90	0.96	0.94
1B	298	181.4	0.61	11.9	121	0.95	0.95
2A	403	218.3	0.54	12.6	132	0.65	0.97
2B	324	234.9	0.73	13	141	0.95	0.95
3A	204	199.6	0.98	13.6	97	0.99	0.96
3B	337	229.7	0.68	14.2	142	0.94	0.9
4A	231	229.3	1.00	21.1	115	0.92	0.96
4B	281	163.7	0.58	18.8	100	0.84	0.97
5A	279	288.2	1.04	15.5	134	1	0.96
5B	280	246.4	0.88	12.1	134	0.99	0.74
6A	172	178.2	1.04	11.1	96	0.99	0.9
6B	253	183.4	0.73	20.8	114	0.9	0.97
7A	292	237.6	0.82	12.2	135	0.99	0.97
7B	144	197.3	1.38	20.9	73	0.94	0.9
Mean	266.4	211.7	0.8	15.4	116.0	0.93	0.93
Total	3729	2964	-	-	1624	-	-
Mean A	258.9	218.1	0.9	14.9	114.1	0.93	0.95
Mean B	273.9	205.3	0.8	16.0	117.9	0.93	0.91
Total A	1812	1526.7	-	-	799	-	-
Total B	1917	1436.8	-	-	825	-	-

SNP number is given for each chromosome. Unique pos.: number of unique positions present for each chromosome. Spearman r with gen. pos.: Spearman’s rank correlation coefficients between DS genetic map and putative genetic positions on bread wheat (BWr). Spearman r with phys. pos.: Spearman’s rank correlation coefficients between DS genetic map and putative physical position. (See text).

Many markers were available for each chromosome, ranging from 144 for chromosome 7B to 403 for chromosome 2A, with an average of 266. This high density genetic map has inter-marker distances ranging from 0.5 to 1.38 cM/marker (chromosomes 2A and 5A, respectively) and an average distance of 0.8 cM between two successive chromosome markers. However, some gaps are still present in the map, especially within chromosome 5A which lacks markers at its distal part, with a huge gap of 15.5 cM. The biggest gap per chromosome is 15 cM long on average.

### Map validation

On average, more than 80% of the markers within any LG shared a common putative assignment on BWr (min of 57% on LG 10 and maximum of 100% for LG 11, S1 Table). Moreover, 86% of the inconsistencies observed between mapping assignments and putative assignment were due to homoeologous competitive-genome assignment (e.g. marker attributed to chromosome 1A instead of 1B). SNPs located in a gene having a very close homeolog could not receive a reliable chromosome assignment, leading to putative chromosome assignment errors. Moreover, if one of the two homoeologous copies of genes was not present in BWr for any reason, the corresponding reads automatically mapped on the corresponding homeo-genome, hence creating confusion. We thus considered, when hesitating between homeologous chromosomes, that the assignment revealed by genetic mapping was more reliable (and thus used) than the putative assignment obtained by blast on BWr.

Some putative chromosome assignment inconsistencies were also observed between non-homeologous chromosomes for 2% of the markers, as summarized in the S2 Table. For example, 11 markers were mapped on chromosome 7A, but they had a putative assignment on chromosome 4A of BWr. The same situation was observed between 4B and 5A (8 markers). The converse situations were also observed (4A-7A and 5A-4B, 7 markers and 4 markers, respectively). These situations always concerned markers grouped together on the genetic map and that had close putative physical positions on BWr (S1 Fig, S2 Table). DWr contigs containing these markers were blasted on the barley genome assembly proposed by the International Barley Genome Sequencing Consortium [49]. Only blasts with similarity higher than 95% were kept, and the best blast hit of each DWr contig was determined using the blastn e-value. Homologous genes were found for 21 of the 30 DWr contigs presenting inconsistencies and were always grouped on the same chromosome. For two of these blocks (LG 23 and 17), durum wheat shared a similar ancestral chromosome assignment with barley, while bread wheat carried a translocation. The reverse situation was observed for the two other blocks (LG 12 and 13) where bread wheat had the same chromosome structure as barley and durum wheat carried translocations (S2 Table).

**Fig 3:**
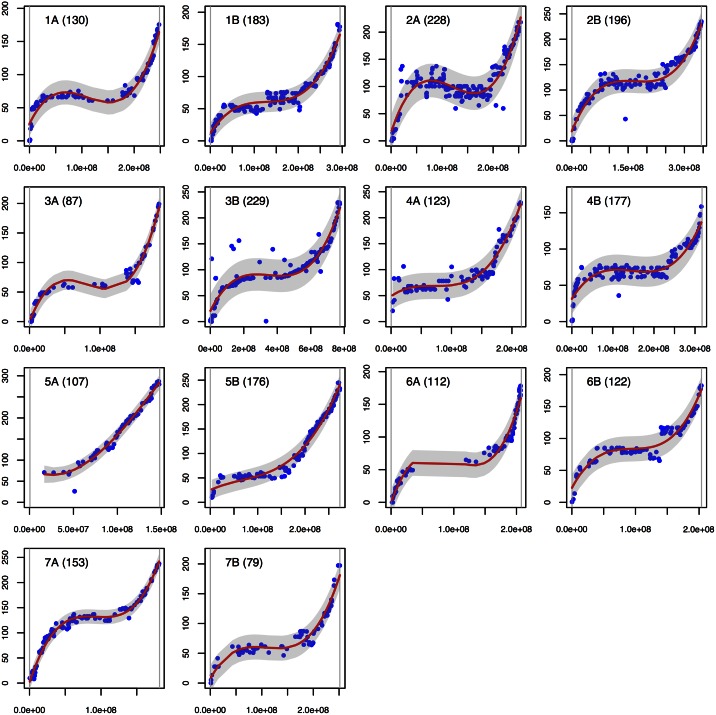
Correlations between putative physical and genetic positions. The 14 durum wheat chromosomes are shown separately, with the putative physical position on the X-axis (bp) and the genetic position on the Y-axis (cM). The chromosome name is given at the top left of each plot. The number of markers is given in brackets. A red line represents the fitted polynomial model and a grey area represents the 95% confidence interval. The two vertical grey lines are maximum and minimum values of the physical positions.

In the vast majority of cases, the marker order was very consistent with physical putative positions (Fig 3). Spearman’s rank correlation coefficients ranged from 0.65 (2A) to 1 (5A), with an average of 0.93. These coefficients were also very high with the genetic positions of the IWGSC zipper, with an average coefficient of 0.93 (min: 0.74; max: 0.97). For each comparison, only one chromosome presented a coefficient of below 0.9, i.e. chromosome 2A when comparing with the putative physical position, and chromosome 5B when comparing with the putative genetic positions. As the Spearman’s rank correlation coefficients between the physical and genetic putative positions themselves were only 0.65 and 0.78 for chromosome 2A and 5A, it may indicate a discrepancy between the genetic (IWGSC zipper) and physical putative positions (POPSEQ data [40]) within the bread wheat reference itself. Our data suggests that the most likely order on the centromeric region of the 2A chromosome is the one proposed by the genetic position of the zipper. This can explain the negative slope of our polynomial regression observed on the centromeric region of the chromosome 2A (Fig 3).

As shown in Fig 3a, markers covered most of the physical chromosome positions except for the middle of chromosomes 6A and 3A, and on the distal part of chromosome 5A, for which markers are missing.

The polynomial regression closely fits the sigmoidal relationship between the genetic and putative physical positions (Fig 3). The adjusted R square ranged from 0.86 to 0.99, with an average of 0.94, and enabled us to identify a few markers outside of the 95% confidence interval (1.7% of the markers on average). Thirty-seven markers were found to be outside of the confidence interval and were thus considered as intra-chromosomal inconsistencies. Chromosome 3B had the highest number of outlier markers (8 markers). Several chromosomes present only one outlier (3A, 5B, 7A, 7B). As many of the outlier markers had multiple blast hits on the bread wheat reference, the inconsistencies could likely be explained by their membership to duplicate/multiple gene families. Consequently, better is to consider their mapping position correct instead of their blast assignment.

## Discussion

### Specific allelic capture is tailored for population genotyping

Genotyping by capture targeted polymorphisms was found to be a powerful tool for characterizing a whole population on the basis of thousands of SNPs. Eighty-five percent of the targeted polymorphic SNPs were recovered, and 65% were of high quality, thus ensuring the relevance of the genetic map. This technique also led to the discovery of untargeted SNPs on homeologous or paralogous genes, which added information on loci that were not formerly detected as polymorphic. The presence of bonus SNPs is an important feature of the genotyping by capture technique since bonus SNPs accounted for 26% of the final number of SNPs (3790).

#### Capture provides fewer but more informative SNPs than arrays

Arrays are currently considered as the most powerful and practical way to genotype a population, but GBS on whole exomes may also become a medium throughput genotyping tool, even for complex species like wheat [22]. Once designed, arrays have major advantages: high-density, simplicity, low missing data rate and no need for labor-intensive bioinformatics treatment. Their main caveats are a lack of flexibility for some applications when used on specific germplasm that differs from the panel upon which the polymorphism was established, for medium density coverage of several thousands of SNPs (e.g. for mapping) and when the cost per individual is an issue.

Working on durum wheat and developing maps on specific parents, genotyping by sequencing targeted captures appeared to be suitable in our case and the obtained results confirmed the efficiency of the technique. The possibility of targeting specific loci had several advantages. First we were able to develop markers on loci known to be polymorphic in our two contrasted parents. This was especially interesting in our case since many SNPs on the 90K iSelect array were monomorphic on durum wheat even though 16 durum varieties were used to screen for polymorphisms [6]. This array was recently used to build a high density consensus framework map [19] based on 10 populations. The number of recovered SNPs per mapping population increased from 2,567 (2.9%) in elite x elite crosses to 10,911 (12.1%) in highly polymorphic crosses involving the emmer wheat *T*. *dicoccum* (S3 Table). Elite x elite populations are much harder to map due to their lack of polymorphism, which could explain the low density of the maps obtained with the 90K iSelect array.

In our case, we targeted 6,240 existing SNPs in the parents and recovered 3790 SNPs (61%), including the bonus-SNP dataset. Roughly comparing the number of SNPs was not sufficient here. As the baits were deliberately designed to sample existing polymorphisms in a maximum of different contigs, it enhanced the mapping accuracy by maximizing the chance of detecting recombination at distant loci. This was clearly noted when comparing the number of unique mapping positions between our map and the 10 durum maps from the 90K iSelect array. The latter maps had between 580 (for elite x elite) and 2,056 unique positions (for emmer x elite), i.e. between 9 and 35% of the total number of markers (S3 Table), while our map was built at 1,624 unique positions for 3,790 markers (43%). As the number of RILs were comparable, even though the number of markers was lower for capture than for the iselect array, it had a higher capacity for spotting distinct loci among chromosomes.

#### Bait captures more than just an SNP

Genotyping by capture provides information on the surrounding genomic context of the targeted SNP markers. Indeed, baits are derived from known contigs, so the surrounding sequence is known for lengths of at least 120 or 200 bp. These surrounding sequences can be highly useful for designing ready to use genome-specific KASPAR markers for breeding.

Confusion between homeo-genomes is a recurrent problem for polyploid species when the divergence between genomes A and B is low. On a targeted position, it may be impossible to unravel the two genomes once captured [21,38,50], leading to loci with a high degree of heterozygosity that are ultimately discarded. Capturing both genomes with genomic surrounding sequences has the double advantage of maximizing the chance of unravelling them and finding new SNPs.

Clearly, a bait can capture untargeted homologous genomic sequences (e.g. homeologs, paralogs). Ideally, this complexity should be addressed by using a complete reference for mapping the reads. Indeed, if the reference contains all existing homologous sequences, then the reads will likely be properly mapped, SNPs adequately identified and genotypes accurately called. In our case, as durum wheat is not sequenced, and as the bread wheat sequence is not fully complete, slightly divergent from durum and contains copy number variations among individuals [22], we preferred to use our DWr reference based on RNAseq [38]. Problems related to undocumented homologous sequences in the reference can lead to excess heterozygosity. In our case, the number of SNPs with excess heterozygous genotypes was very low (3%), which confirms the quality of our DW reference and that 120 bp are sufficient for overcoming most homolog-related ambiguity.

#### SNP capture is cheap and easy to handle

Exon capture is efficient for deciphering gene complexity in wheat [32] but still cannot be used routinely to genotype large populations at a reasonable cost. To this regard, using capture on restricted genomic fragments could bring efficient complementary tools for genotyping relatively large populations at reasonable cost. Bioinformatics treatments are facilitated by the marked reduction in genome complexity due to the low percentage of genomes actually sequenced. The cost of SNP capture could thus become very attractive in situations where either DNA arrays are not available or not adapted (medium size and medium throughput situations), and since whole exome capture remains unaffordable for specific purposes such as mapping or genomic selection.

Our entire protocol is based on the possibility of buying bait batches (we used the minimum purchase of 20,000 baits, http://www.mycroarray.com/) for a particular program, allowing the capture of 576 (12×48) genomic libraries in our current conditions. The cost of these individual libraries, already optimized [46], is still around €10. Sequencing a multiplex of 192 captured libraries on a single lane of Hiseq3000 Illumina sequencer puts the final cost of data production at about €28–30 per genotype in our conditions. By data point, the cost could appear more expensive than genotyping by sequencing (GBS) or microarrays, but GBC on targeted polymorphisms is more accurate than GBS in complex species like durum wheat, and does not require the initial investments for a micro-array development. Furthermore, GBC allows for a very quick adjustment between individuals number and SNP number, i.e., increasing multiplexing and reducing the number of targeted genes, and it does not depend on a specific platform to call the genotypes.

This study showed that only two 120 bp baits were enough to capture a locus. This makes future genotyping experiments targeting 10,000 SNPs possible, therefore considerably decreasing the price per data point. Moreover, genotyping by capture was found to be an appropriate tool for maximizing pooling for sequencing. The main caveat in our experiment concerned the large number of reads mapping in microsatellite-rich regions. We expect that substantial progress could be achieved in this respect, hence significantly increasing the number of effective reads, by using of an ad’hoc blocking DNA and avoiding to design baits in microsatellite rich contigs.

Working on other durum wheat populations (including elite x elite) will help assemble a database of tested useful baits targeting 10,000 SNPs located in as many contigs. This set could be used to score very large populations for few SNPs by selecting only the most relevant baits for the task, hence allowing drastic increases in multiplexing. It could also be used for targeting 10,000 or more SNPs on few individuals.

### High quality genetic map

Genotyping by capture of targeted loci enabled us to obtain a dense high quality genetic map of 3,729 markers containing 1,624 unique positions.

#### Highly dense SNP capture genetic map

Our map has almost as many markers as individual maps obtained by the 90K iSelect array [6], but provides about twofold more unique positions per SNP. The coverage is similar (0.8 cM between adjacent unique positions) to that of very dense individual maps recently published [19,51]. Our DS map (2,964 cM) is very similar in length to other cultivated durum emmer maps (2,635 cM, [19]), but slightly longer than SNP-based maps, including that of wild wheat (2,258 cM, [51]). As genes are not evenly distributed along chromosomes, some gaps between adjacent markers may be explained by the tendency of our transcript-derived SNPs to be enriched in some areas while lacking density in some others [19,39]. For example, the lack of markers at some distal positions of the map may be due to the fact that some blocks of heterochromatin regions have few transcribed sequences [39].

#### SNP capture genetic map is highly consistent with the BW genome

The putative assignment of markers on BWr was highly consistent with LG formation. However, some markers were highly similar to a BWr chromosome while being in strong LD with many markers assigned to a different BWr chromosome. Such chromosome assignment swaps occurred between loci of chromosomes 7A-4A and 4B-5A (S1 Fig). The long arms of chromosomes 4A and 5A, and the short arm of chromosome 7B of Chinese Spring have already been reported to be involved in interchange. Chromosome 4A also underwent paracentric and pericentric inversions [52,53]. Durum wheat synteny with barley has also been better shown on a *durum* x *dicoccoides* cross when markers of 4A/5A/7B translocations were removed [51]. Here we found that a translocation involving 4A/7A and 4B/5A chromosomes in bread wheat were actually non-translocated in durum compared to barley, while the reciprocal situation was observed for 5A/4B and 4A/7A translocations. As emmer wheat exhibits a somewhat high level of translocation polymorphism [54], such translocation differences between durum and bread wheat may be the result of differential fixation of translocations, even though bread wheat seems to have a free-threshing *turgidum* ancestor [55]. Durum and bread wheat thus differ in a small but significant proportion of some of their chromosomes.

#### Recombination rate logically increases in distal parts of the 3B chromosome

As the 3B chromosome of BWr is fully sequenced, the physical positions are much more precise, which permits accurate estimation of the recombination rate for this chromosome. The recombination pattern in DS was very similar to that observed by Maccaferri et al. in durum wheat [19]. First, in the two distal regions, corresponding to [0–61.6] cM in 3BS and [133.4–210.8] cM in 3BL on the durum consensus map, the physical-to-genetic distance relationship was mostly linear ([19], Fig 3) and corresponded to distal recombination rates of 0.68 cM/Mb in 3BS and 0.87 cM/Mb in 3BL, respectively. On these two segments, corresponding to the [0–90] Mb and [686–774] Mb physical segments of the 3B pseudo-molecule, the recombination rates in DS were 0.55 (3BS) and 0.73 cM/Mb (3BL). Secondly, the two maps gave a very close and low recombination rate in the 200–600 Mb pericentromeric segment, i.e. 0.06 cM/Mb in DS and 0.07 cM/Mb in the tetraploid consensus map ([19], Fig 3). These two observations suggest an increase in recombination intensity at the distal end of 3BL, as already described [39].

## Conclusion

Our study demonstrates the feasibility of a genotyping by capture approach for complex polyploid species such as durum wheat, and confirmed that it is a reliable strategy for genotyping whole populations for thousands of SNPs. The set of baits required to genotype this population is provided with a real effort made to localize the proposed markers. These resources will likely be useful for other mapping populations including a *T*. *dicoccum* parent. The capture efficiency could also be improved by targeting more loci and taking care concerning the bait area in order to avoid the presence of microsatellite repetitive elements.

Genotyping by capture could be used to easily target durum elite specific polymorphisms. Nevertheless it is still hard to predict if seeking polymorphism in coding sequence (using RNAseq as has been done here or in the 90K iSelect array) will be sufficient to get polymorphism in area deeply depressed by the successive bottlenecks experienced by elite durum [56,57]. In this case, baits could also be based on polymorphism identified in non-coding genomic sequences and already tested on microarrays such as the Breedwheat Axiom 420 K (www.breedwheat.fr/). As whole exome capture is developing, preliminary sequencing of a set of interesting parents followed by the development of specific baits would help to rapidly identify recombinants in large population sets or to map efficiently interconnected mapping populations.

## Supporting Information

S1 Fig Correlations between putative physical and genetic positions. The 14 durum wheat chromosomes are shown on the same plot, with the putative physical position of SNPs on the X-axis (bp) and the genetic position on the Y-axis (cM). It allows checking for markers having distinct genetic and putative attributions.
(PDF)

S1 File Durum Wheat reference transcriptome (DWr). The fasta file of the *de novo* assembly obtained from [38].
(GZ) Click here for additional data file.

S2 File Detailed capture protocol. Description of the exact protocol used for the capture step.
(DOC)

S3 File Method used to map low covered SNPs. This file explains how we estimated the genetic positions of SNPs that were genotyped for fewer than 100 individuals but more than 50.
(DOCX)

S4 File Genetic map of the *Dic2* x *Silur* population. The genetic map is provided here. Markers names follow the nomenclature A@pos, with A being the contig name in the DWr [38] and pos being the position of the SNP in this contig. The positions are given in cM. SNP type can be either “mapped” or “DL_mapped” if less than 100 individuals were genotyped (see S4 File).
(CSV)

S5 File Fasta file containing the bait sequences. The set of baits used for this study is provided. Baits suspected to capture microsatellite-rich regions were removed. Bait names are as follows: A@pos|B|C|D|E with:
A: contig name in the DWr (S1 File)pos: position of the polymorphism previously detected in the RNA-seq experiment between dic2 and silur in the contig A.B: parental allelic status of the bait (Dic2 or Silur)C: position of the SNP in the baitD: type of bait (type 1, 2 or 3)E: origin of the SNP (DS or EPO population, see text)
(GZ) Click here for additional data file.

S1 Table Putative assignment of markers composing linkage groups. The characteristics of the 25 linkage groups (LG) are presented. For each LG, contigs containing markers are distributed on the 14 A+B chromosomes of the IWGSC reference according to their best blast score (physical putative assignment). For each LG, the percentage of markers with a consistent genetic and putative assignments on the same chromosome are given, as well as the percentage of markers distributed on homeologs and different chromosomes.
(XLSX)

S2 Table Analysis of 7A-4A and 5B-4A inconsistencies. Thirty markers showed inconsistency between the genetic and putative assignments to chromosomes. Their names, barley and IWGSC assignments (bp) and DS genetic positions (cM) are reported. Assignments were obtained by blast on barley [49] and IWGSC. The corresponding barley gene IDs are given when available, as well as their corresponding chromosomes. The physical positions on barley were taken from: Mascher M, Muehlbauer GJ, Rokhsar DS, Chapman J, Schmutz J, Barry K, et al. Anchoring and ordering NGS contig assemblies by population sequencing (POPSEQ). Plant J. 2013;76: 718–27.
(XLSX)

S3 Table Description of 10 durum wheat genetic maps. Among the populations used to build the consensus durum wheat map published by Maccaferri et al. [19], 10 have been genotyped on the 90K iSelect array [6]. The features of these maps are presented here and compared with the genotyping by capture approach.
(XLSX)
