# Supplementary figures and images for: Genotyping by Sequencing Using Specific Allelic Capture to Build a High-Density Genetic Map of Durum Wheat
Source: PLoS One. 2016 May 12;11(5):e0154609. doi: 10.1371/journal.pone.0154609 (PMC4865223; doi:10.1371/journal.pone.0154609)

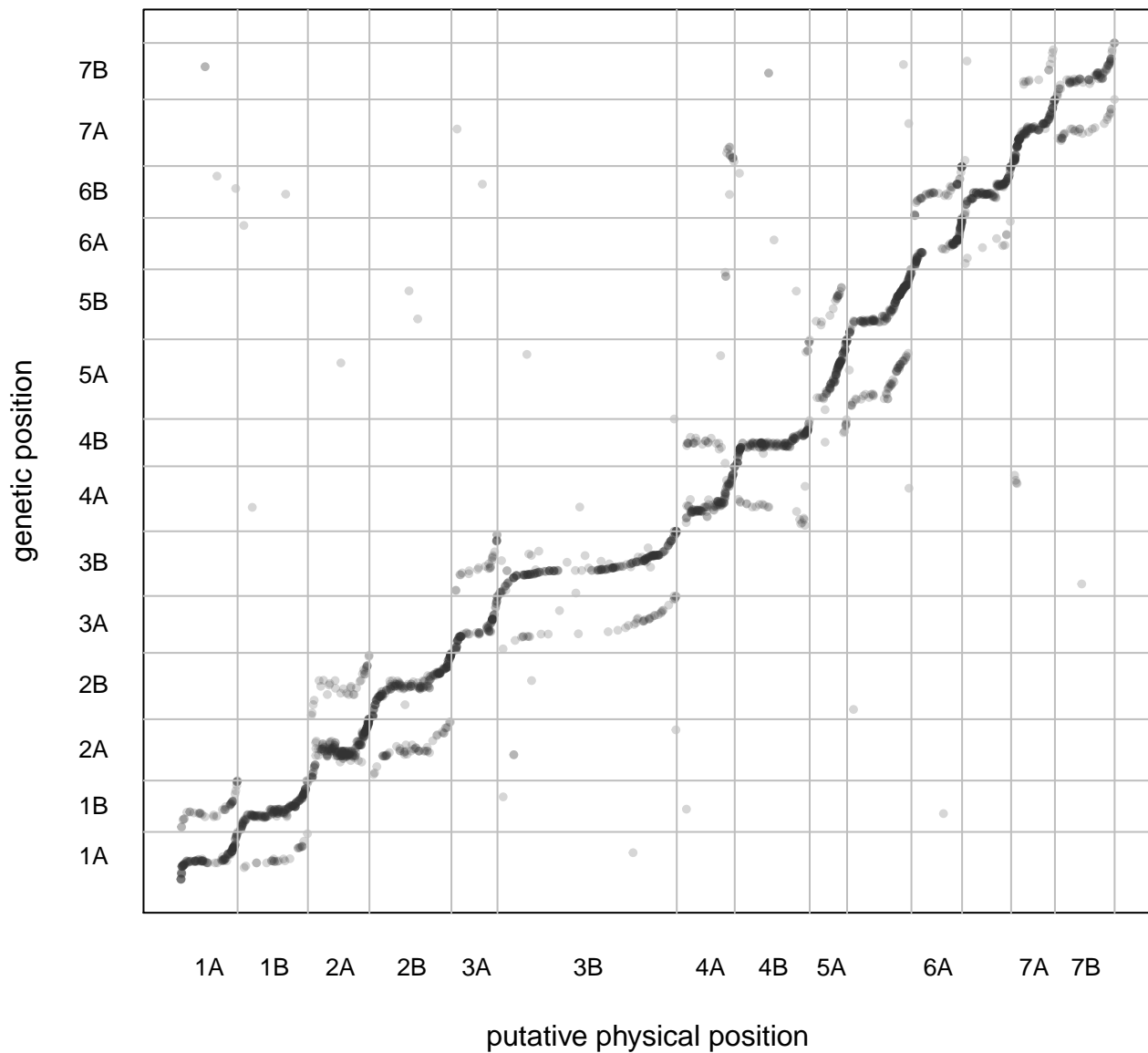

Supplement: S1 Fig — The 14 durum wheat chromosomes are shown on the same plot, with the putative physical position of SNPs on the X-axis (bp) and the genetic position on the Y-axis (cM). It allows checking for markers having distinct genetic and putative attributions. (PDF) [file pone.0154609.s001.pdf]
