# Supplementary material for: Genotyping by Sequencing Using Specific Allelic Capture to Build a High-Density Genetic Map of Durum Wheat
Source: PLoS One. 2016 May 12;11(5):e0154609. doi: 10.1371/journal.pone.0154609 (PMC4865223; doi:10.1371/journal.pone.0154609)
Supplement: S2 File — Description of the exact protocol used for the capture step. (DOC) [file pone.0154609.s003.doc]

**S2 File**. **Detailed capture protocol.**

**Plant DNA purification**

DNA was extracted from 100 mg of fresh young leaves with the Chemagic DNA Plant Kit (Perkin Elmer), according to the manufacturer’s instructions. The protocol is adapted to the use of the KingFisher Flex™ (Thermo) automated DNA purification workstation.

# Construction of enriched library and sequencing

Library preparation for multiplexed individuals follows the published protocol of Rohland and Reich, 2012, with some modifications.

**1° Target preparation, construction of barcoded genomic libraries**

*Step 1 : For each individual, 1 µg of total DNA (in 100 µL of water) are sheared using a Bioruptor Pico sonication device in 500 µl microtubes to a targeted 300 bp DNA fragment size using parameters of the 300pb standard protocol for DNA shearing. (*[*https://www.diagenode.com/files/protocols/Standard_protocols_for_DNAShearing.pdf*](https://www.diagenode.com/files/protocols/Standard_protocols_for_DNAShearing.pdf)*).*

Step 2: Dual fragment size selection (DFSS) is done using Agencourt AMPure XP magnetic beads (Beckman Coulter, Brea, CA 92821, cat. No. A63880) by adding 0.7x volume of AMPure XP to 70µl of fragmented DNA, mixed and incubated for 10 minutes. After the beads are discarded an additional 0.25x AMPure XP volume is added to the supernatant, mixed and incubated for 10 minutes. Supernatant was removed and two 70% ethanol-washes are done. Air dried beads were resuspended in 50 µl TE 1X buffer.

Step 3: DNA after DFSS are blunted and 5’ phosphorylated using the Thermo Scientific Fast DNA End Repair Kit (Thermo Fischer Scientific , K0771).

Step 4: Fragmented and repaired DNA are individually controlled (sizing and estimation of the concentration) by electrophoresis on a Fragment Analyzer™ (AATI) device with the DNF-474 High Sensitivity Fragment Analysis Kit.

Step 5: 50 ng of fragmented DNA are ligated with 8 pmol of PE-P5 and PE-P7 adapters. Each PE-P5 adapter carries a specific hexamer barcode . Reactions are conducted in 15 µl final volume with 1 unit of T4 DNA ligase for 1 hour at 22 °C followed by a heat inactivation step at 65°C for 10 minutes.

Step 6: 48 samples (corresponding to 48 hexamer barcodes on the PE-P5 adapter) (Rohland and Reich, 2012) are pooled. A clean-up step is performed with 1.8x volume of AMPure XP magnetic beads. The elution volume is 96 µL.

Step 7: A nick fill-in step is performed using 64 units of Bst DNA polymerase (New England Biolabs, M0275), 1x ThermoPol reaction buffer, 250 µM dNTP in 120 µl final volume and incubated for 15 minutes at 37°C. A clean-up step is performed with 1.8x volume of AMPure XP magnetic beads. The elution volume is 40 µL.

Step 8: For each pool of 48 samples, a pre-hybridization PCR is performed using the Phusion® High-Fidelity PCR Master Mix (Thermofischer, 1040-2678) with 200 nM PreHyb-PE_F (ctttccctacacgacgctcttc) and 200 nM PreHyb-MPE_R (TGACTGGAGTTCAGACGTGTG) primers in a final volume of 100µl.

Thermocycling parameters: 3 minutes at 8°C, followed by 12 cycles of 80 seconds at 98°C; 45 seconds at 55°C and 60 seconds at 68°C, with a final elongation of 10 minutes at 72°C. A clean-up step is performed with 1.8x volume Agencourt AMPure XP magnetic beads. The elution volume is 10 µL.

**2° Enrichment, capture by hybridisation**

**The protocol used is based on the User Manual of the MYBaits Sequence Enrichment for Targeted Sequencing kit (http://www.mycroarray.com/pdf/MYbaits-manual-v2.pdf).**

*Step 9: For each pool of 48 barcoded libraries, in solution capture is carried out in a 40 µl final with 500 ng of mixed libraries and 100 ng of biotinylated RNA probes (5.5 µL of MYBaits Capture Probe). Hybridization was performed in a thermocycler (Biometra T1) during20 hours at 65°C after an initial denaturation cycle at 95°C for 5 minutes..*

Step 10: Biotinylated probes annealed to the target complexes are then immobilized with 25 µl of Streptavidin coupled Dynabeads C1 (Invitrogen, 650.01) at 5 µg/µl in a 2X binding and washing buffer (BW) and resuspended and washed as recommended by the manufacturer. Immobilization is conducted in 80 µl final volume with 1X BW buffer and is placed for 1 hour at 45 °C. Samples are then placed in a magnet plate and supernatant is removed.

Step 11: Beads are then subject to one wash with 130 µl of 1X SSC/0.1% SDS for 5 minutes at 65 °C followed by three 5-minutes washes at 65°C with 0.1X SSC/0.1% SDS and a final wash with 0.2X SSC for 5 minutes at room temperature.

Step 12: Captured DNA is then eluted with 15 µl H_2_O by incubation at 80°C for 30 minutes. Samples are placed in a magnet plate and the supernatant which contains the hybridized products are retained.

**3° PCR post-capture and sequencing**

Step 13: A PCR amplification is undertaken to enrich library fragments, extend the adaptor sequence and incorporate an index to the P7 adaptor. The PCR reaction is using the Phusion® High-Fidelity PCR Master Mix (Thermofischer, 1040-2678) with 500 nM SOL-PE-PCR_F (aatgatacggcgaccaccgagatctacactctttccctacacgacgctcttc) (Rohland and Reich, primer and 500 nM SOL-MPE-INDX_R (CAAGCAGAAGACGGCATACGAGATXXXXXXGTGACTGGAGTTCAGACGTGT) indexed primers . This primer carries 6 bases of the official Illumina Index. Thermocycling parameters: 2 minutes at 98°C, followed by 15 cycles of 20 seconds at 98°C; 30 seconds at 62°C and 30 seconds at 72°C, with a final elongation of 5 minutes at 72°C. The reaction volume is 50 µL. A clean-up step is performed with 1.8x volume Agencourt AMPure XP magnetic beads. The elution volume is 20 µL.

Step 14: Indexed libraries are individually controlled (sizing and estimation of the concentration) by electrophoresis on a Fragment Analyzer™ (AATI) device with the DNF-474 High Sensitivity Fragment Analysis Kit.

Step 15: Four indexed libraries, corresponding to 192 captured barcoded DNA samples, are equally mixed. The final pooled library is quantify by qPCR with the KAPA Library Quantification Kit and provided to the Get-PlaGe core facility (GenoToul platform, INRA Toulouse, France http://www.genotoul.fr) for sequencing.

Step 16: The final pooled library is sequenced using the Illumina paired-end protocol on a single lane of a HiSeq3000 sequencer, for 2 x 150 cycles.

References

Rohland N, Reich D (2012) Cost-effective, high-throughput DNA sequencing libraries for multiplexed target capture. *Genome Research* **22**, 939-946.
