# Supplementary material for: Genotyping by Sequencing Using Specific Allelic Capture to Build a High-Density Genetic Map of Durum Wheat
Source: PLoS One. 2016 May 12;11(5):e0154609. doi: 10.1371/journal.pone.0154609 (PMC4865223; doi:10.1371/journal.pone.0154609)
Supplement: S3 File — This file explains how we estimated the genetic positions of SNPs that were genotyped for fewer than 100 individuals but more than 50. (DOCX) [file pone.0154609.s004.docx]

**S3 File: Method used to map low covered SNPs.**

To position the maximum number of markers on the map, 280 SNPs for which the number of genotyped RILs was comprised between 50 and 99 were positioned. We estimated their genetic position by seeking in the mapped markers set those that had the maximum value of linkage disequilibrium measured by the r² given by regressing allelic frequencies by pair of SNPs (42).

When many markers gave the same maximum values for r² with a candidate SNP, we first verified that they belonged to the same chromosome and then estimated the cM value by the average of map positions of the linked mapped markers. These markers are provided in the S4 File but are not taken into account for building the map. All of them but one were affected unambiguously to a chromosome or a small LG.
